# A comprehensive analysis of drug resistance molecular markers and *Plasmodium falciparum* genetic diversity in two malaria endemic sites in Mali

**DOI:** 10.1186/s12936-019-2986-5

**Published:** 2019-11-12

**Authors:** Seidina A. S. Diakité, Karim Traoré, Ibrahim Sanogo, Taane G. Clark, Susana Campino, Modibo Sangaré, Djeneba Dabitao, Antoine Dara, Drissa S. Konaté, Fousseyni Doucouré, Amadou Cissé, Bourama Keita, Mory Doumbouya, Merepen A. Guindo, Mahamoudou B. Toure, Nafomon Sogoba, Seydou Doumbia, Gordon A. Awandare, Mahamadou Diakité

**Affiliations:** 1 Malaria Research and Training Center, University of Sciences, Technics and Technologies of Bamako (USTTB), Bamako, Mali; 2 West African Centre for Cell Biology of Infectious Pathogens, University of Ghana, Accra, Ghana; 3 Faculty of Infectious and Tropical Diseases, London School of Hygiene and Tropical Medicine, London, UK

**Keywords:** *Plasmodium falciparum*, Drug-resistance, Molecular surveillance, Next-generation sequencing

## Abstract

**Background:**

Drug resistance is one of the greatest challenges of malaria control programme in Mali. Recent advances in next-generation sequencing (NGS) technologies provide new and effective ways of tracking drug-resistant malaria parasites in Africa. The diversity and the prevalence of *Plasmodium falciparum* drug-resistance molecular markers were assessed in Dangassa and Nioro-du-Sahel in Mali, two sites with distinct malaria transmission patterns. Dangassa has an intense seasonal malaria transmission, whereas Nioro-du-Sahel has an unstable and short seasonal malaria transmission.

**Methods:**

Up to 270 dried blood spot samples (214 in Dangassa and 56 in Nioro-du-Sahel) were collected from *P. falciparum* positive patients in 2016. Samples were analysed on the Agena MassARRAY^®^ iPLEX platform. Specific codons were targeted in *Pfcrt*, *Pfmdr1*, *Pfdhfr*, and *Pfdhps, Pfarps10, Pfferredoxin, Pfexonuclease* and *Pfmdr2* genes. The Sanger’s 101-SNPs-barcode method was used to assess the genetic diversity of *P. falciparum* and to determine the parasite species.

**Results:**

The *Pfcrt*_76T chloroquine-resistance genotype was found at a rate of 64.4% in Dangassa and 45.2% in Nioro-du-Sahel (*p *= 0.025). The *Pfdhfr_51I*-*59R*-*108N* pyrimethamine-resistance genotype was 14.1% and 19.6%, respectively in Dangassa and Nioro-du-Sahel. Mutations in the *Pfdhps_S436*-*A437*-*K540*-*A581*-*613A* sulfadoxine-resistance gene was significantly more prevalent in Dangassa as compared to Nioro-du-Sahel (*p *= 0.035). Up to 17.8% of the isolates from Dangassa *vs* 7% from Nioro-du-Sahel harboured at least two codon substitutions in this haplotype. The amodiaquine-resistance *Pfmdr1*_N86Y mutation was identified in only three samples (two in Dangassa and one in Nioro-du-Sahel). The lumefantrine-reduced susceptibility *Pfmdr1_Y184F* mutation was found in 39.9% and 48.2% of samples in Dangassa and Nioro-du-Sahel, respectively. One piperaquine-resistance *Exo_E415G* mutation was found in Dangassa, while no artemisinin resistance genetic-background were identified. A high *P. falciparum* diversity was observed, but no clear genetic aggregation was found at either study sites. Higher multiplicity of infection was observed in Dangassa with both COIL (*p *= 0.04) and Real McCOIL (*p *= 0.02) methods relative to Nioro-du-Sahel.

**Conclusions:**

This study reveals high prevalence of chloroquine and pyrimethamine-resistance markers as well as high codon substitution rate in the sulfadoxine-resistance gene. High genetic diversity of *P. falciparum* was observed. These observations suggest that the use of artemisinins is relevant in both Dangassa and Nioro-du-Sahel.

## Background

Despite numerous advances in malaria control strategies, the disease still kills countless children worldwide, mainly in sub-Saharan African countries. The World Health Organization (WHO) reported no significant progress in reducing global malaria burden during the period from 2015 to 2017 [1]. In Mali, as well as in the rest of the world, resistance to anti-malarial drugs is one of the greatest challenges of the National Malaria Control Programme (NMCP) [2, 3]. High prevalence of resistance to chloroquine and sulfadoxine-pyrimethamine (SP) led the Malian NMCP to switch to artemisinin- based combination therapy (ACT) in the 2000s, as recommended by the WHO [4]. Artemisinin and its derivative drugs (Artemisinins) are still very effective in Africa [5, 6]. Resistance to these drugs have been reported in East-Asia [7, 8]. One of the rationales of ACT use is to maintain long-term efficacy of artemisinins to *P. falciparum* [4]. To achieve this objective, the choice of the artemisinin partner drugs is important and should be made based on their effectiveness on circulating local parasite isolates. Consequently, it appears important to monitor the emergence and spread of the anti-malarial drug resistance in different geographical and endemic areas in order to inform the choice of the anti-malarial molecules to be associated with artemisinins in ACT [9, 10]. Also, assessing the genetic diversity of *P. falciparum* populations in different regions may allow to tract malaria parasites circulating across different geographic areas.

Molecular markers of drug-resistance are very useful in identifying drug-resistant *P. falciparum.* They are used in epidemiological surveillance of drug-resistance including their emergence and spread monitoring. Molecular markers have been described for many of the common anti-malarial drug and are constituted of either single nucleotide substitution (SNP) or concatenated SNP in genes involved in parasite interaction with drugs. Evidence of association between several alleles in *P. falciparum* multi-drug resistance (*Pfmdr*) gene and chloroquine-resistance was reported in 1990 [11]. However, the K76T mutation in *P. falciparum* chloroquine resistance transporter gene (*Pfcrt_K76T*) mostly associated with the *Pfcrt*_72C-73V-74I-75E-76T haplotype in Africa is known as the strongest chloroquine-resistance marker in *P. falciparum* [12–15]. Resistance to pyrimethamine has also been associated with numbers of mutations combination in the *P. falciparum* dihydrofolate-reductase (*Pfdhfr*) gene, such as the *Pfdhfr*_51I-59R-164L [16] and *Pfdhfr*_51R-59N-108I genotypes [17, 18]. Also various combinations of substitutions at the codons 436, 437, 540, 581 and 613 of the *P. falciparum* dihydropteroate-synthetase (*Pfdhps*) gene may confer resistance to sulfadoxine [19]. The greater the number of substitution accumulation is in the gene, the more the parasite gets resistant to the sulfadoxine. The *Pfdhps*_437G-540E genotype was associated with substantially decreased susceptibility to sulfadoxine. In vitro experiments have shown that N86Y mutation in the *P. falciparum* multidrug resistance-1 (*Pfmdr1)* gene (*Pfmdr1_86Y)* increases the fifty percent inhibitory concentrations (IC50) of chloroquine and amodiaquine [20, 21].

Mutations in multidrug resistance protein (*pfmdr1*) have been associated with various parasite response to mefloquine and lumefantrine [22]. Reduced susceptibility to lumefantrine has been associated with the *Pfmdr1_*Y184F mutation [23–25]. In a recent genome-wide association study (GWAS), the E415G mutation, a SNP in *P. falciparum* exonuclease gene (*Pfexo_E415G)*) was associated with ex vivo piperaquine IC50 of parasite isolates from Cambodia [26]. The co-inheritance of several mutations in *P. falciparum* Apicoplast Ribosomal Protein S10 (*Pfarps10)* gene [*Pfarps10_*127M-128Y/H], the P*. falciparum* ferredoxin (PfFd) gene [*Pffd_ 193Y]*, the PfCRT gene: [*Pfcrt*_326S-356T], and the PfMDR2 [*Pfmdr2_T484I*] were shown to constitute a genetic background that allows for the emergence of mutations in the *P. falciparum kelch 13* gene [27], a known artemisinin resistance locus [28–30]. This haplotype is referred to as artemisinin-resistant parasite genetic background (Art-resistant PGB).

All thought previous studies has provided insights into some anti-malarial drug resistance markers in the south of Mali [31, 32], the risk of *P. falciparum* infection is variable from south (with intense and long transmission) to north (with unstable and short transmission) in Mali. This variability is related to environmental and climatic conditions. The level of transmission may also impact the malaria parasite genetic diversity and the spread of resistance to anti-malarial drugs. In fact, the higher the malaria transmission is, the more diverse the parasite population is, the more likely the parasite gets resistant to anti-malarial drugs.

In this study, both the genetic diversity of *P. falciparum* and the distribution of drug resistance markers were assessed in two distinct eco-climatic areas in Mali.

## Methods

### Study sites

The epidemiology, entomology and the impact of malaria vary widely across the Malian territory [33, 34]. The study was carried out in Dangassa (Lat = 12.150,925, Long = − 8.206974) and Nioro-du-Sahel (Lat = 15.224674, Long = − 9.583888). Dangassa is located in south savannah grassland of Mali along the right side of Niger River and has year-round access to water (Niger River). Malaria transmission is seasonal and mainly occurs from June to December. However, dry season transmission may occurs because of the Niger River. Indeed, mosquito breeding occurs primarily in microhabitats, such as the footprints of cattle and the receding river in the dry season, which leaves natural pools as well as man-made pools from river gold mining. Nioro-du-Sahel is located in Sahelian region of Mali near the Islamic Republic of Mauritania. Malaria transmission is unstable and occurs from July to September. *Anopheles gambiae* breeding sites are strictly rain-dependent. *Anopheles gambiae* (about 95%) and *Anopheles arabiensis* (about 5%) are the predominant malaria vectors [35] in the two sites. Population migration occurs in the two localities mainly for gold mining for Dangassa and for herding or for trading for Nioro-du-Sahel. In both localities, malaria control strategies rely on the use of long-lasting insecticide-impregnated nets (LLINs), ACT for treatment and sulfadoxine-pyrimethamine (SP) for intermittent preventive treatment of pregnant women (IPTp). In addition, the population may have access to other malaria drugs in the local market in the two localities.

### Study design and sample collection

The study has been set up by the research team in collaboration with the local health staff for the entire malaria transmission season (June to December 2016). A medical team was installed at the health centre of the village. Population of all age group was encouraged to attend the local health centre if febrile, where they were examined by a study physician. Malaria cases were diagnosed by a rapid diagnostic test (RDT) [CareStart™: Malaria HRP2/pLDH(Pf/PAN) Combo] and/or microscopy. A four (4) ml venous blood was obtained from each TDR positive patient. A dried blood spot (DBS) was made by dropping approximatively 50 μl of the venous blood on a filter paper. The DNA was extract from the DBS and used for *P. falciparum* genomic and host genetic studies. Confirmed malaria cases were classified as mild or severe according to the World Health Organization (WHO) definitions [36], and treated according to the Malian NMCP guidelines (oral ACT for uncomplicated malaria, intramuscular (i.m) administration of artemether and/or quinine for severe malaria).

### DNA extraction, sample processing, and genotyping

The dried blood spots on filter paper were sent to the Wellcome Sanger Institute for processing. DNA extraction was carried out using the Qiagen DNA Investigator Kit (No. 56504, Qiagen, Crawley, UK) for high-throughput robotic processing. DNA was eluted in 100 μl TE buffer and stored at − 20 °C for later use. Extracted DNA underwent whole genome amplification (WGA) by primer-extension pre-amplification [37] or selective WGA [38] prior to genotyping. Genotyping was performed according to manufacturer’s instruction on the Agena MassARRAY^®^ iPLEX platform (Agena Bioscience, Hamburg, Germany). This system is able to accurately genotype large numbers of samples for multiple SNPs simultaneously. It previously been used in *P. falciparum* for sequencing validation of novel SNPs [39]. All genotypes were called from background adjusted peak intensities, which were normalized and called by batch. In brief, batches underwent calling using a heuristic algorithm which identifies intensity ranges for each SNP in single infection samples, and called mixed base loci (“heterozygous”) based on those range thresholds, adjusting for background intensity. Batches were plate based and contained between 96 and 384 samples. These are necessary to generate per assay ranges of intensities to calculate background intensity levels.

### Parasite diversity and multiple infection assessment

The malaria parasite diversity and multiplicity of infection were assessed using the Sanger’s *P. falciparum* barcodes formed by concatenated genotypes at 101 SNPs across *P. falciparum* genome. These SNPs are all bi-allelic, at low to medium frequency in global parasite populations, and were chosen for their usefulness in analyses of relationship between *P. falciparum* parasites. They have been used previously to estimate levels of relationship and importation [40] as well as complexity of infection. Multiplicity of infection estimates were produced by both the programmes COIL [41] using default parameters and The Real McCOIL [42] using the bi-allelic SNP barcodes.

### Drug resistance markers assessment

Specific mutations in *P. falciparum* genome were screened using the Agena MassARRAY^®^ iPLEX platform as above. The following codons in the *P. falciparum* genome were screened for substitution: K76, N326, I356 in the *Pfcrt* gene, the N86, Y184, D1246 in the *Pfmdr1* gene, the N51, C59, S108, I164 in the *Pfdhfr* gene, the S436, A337, K540, A581, A613 in *Pfdhps* V127, D128 in the *Pfarps10* gene, the D193 in *Pf*-*ferredoxin* gene, the E415 in *Pf*-*exonuclease* gene, and T484 in *Pfmdr2.* Single isolate mutations or combination of mutations in these codons are associated with resistance to anti-malarial drugs as specified in the background section.

### Statistical analysis

This was an inclusive study. All the confirmed malaria case detected during the study period in the two sites were enrolled. A Chi square test was used to compare the prevalence of molecular markers in the two study sites. When the effective ≥ 30, the Z-test was considered. The significant P value was set as equal or inferior to 0.05.

For the Real McCOIL determination, the categorical method was used with the following parameters, maxCOI = 25, threshold_ind = 20, threshold_site = 20, totalrun = 10,000, burnin = 1000, M0 = 15, e1 = 0.05, e2 = 0.05, err_method = 1. Principal component analysis was performed to assess and compare the parasite diversity between the two study sites.

## Results

**Fig. 1:**
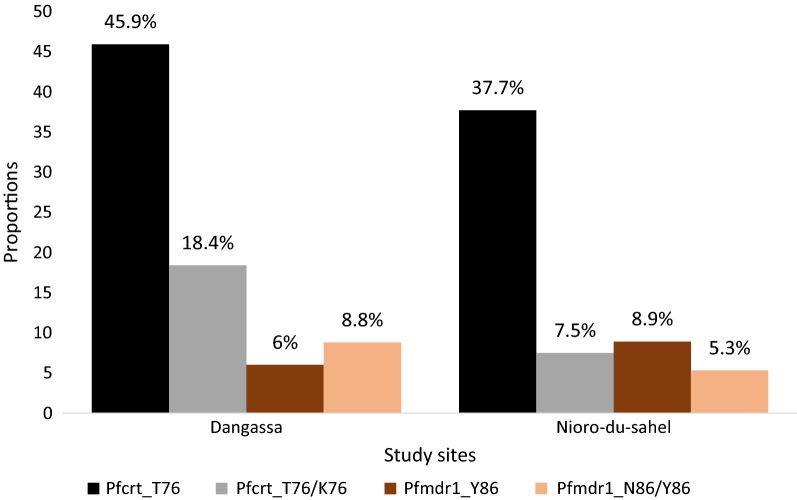
Proportion of P. falciparum isolates harbouring Pfcrt_K76, Pfcrt_76T and Pfmdr1_86Y genotypes

**Fig. 2:**
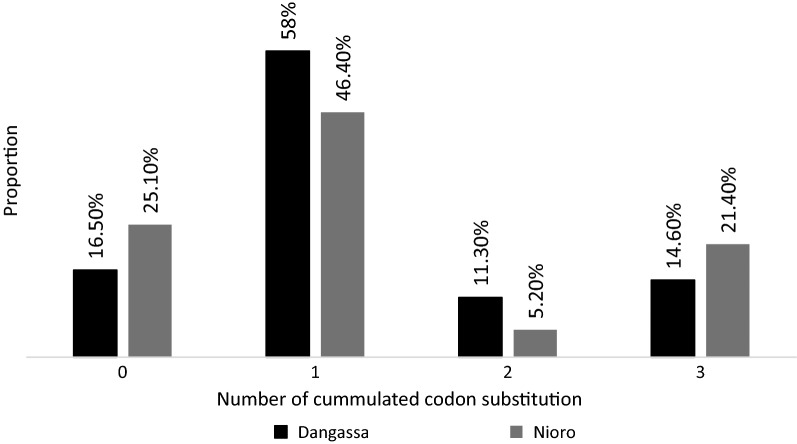
Proportion of number of accumulated codon-substitution in *Pfdhfr* gene at positions 51, 59, 108 and 164 in Dangassa and Nioro-du-sahel

**Fig. 3:**
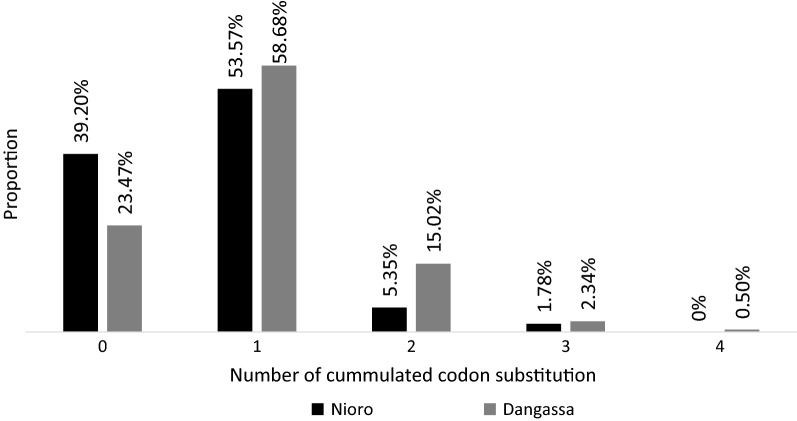
Proportion of number of accumulated codon-substitution in *Pfdhps* gene at positions 436, 437A, 540, 581 and 613 in Dangassa and Nioro-du-sahel

In total, 270 *P. falciparum* samples (214 from Dangassa and 56 from Nioro-du-Sahel) were analysed. The analysis of anti-malarial drug genes showed very high rates of mutations. The chloroquine-resistance *Pfcrt*-76T genotype was significantly more prevalent in Dangassa [64.4% (45.9% 76T + 18.4% 76K/76T)] compared to Nioro-du-Sahel [45.2% (37.7% 76T + 7.5% 76K/76T)] (p = 0.025) (Fig. 1). This study revealed a very high but similar rate of codon-substitution in the pyrimethamine-resistance *Pfdhfr* gene at positions 51, 59, 108 and 164 in both Dangassa and Nioro-du-Sahel (Fig. 2) (p = 0.2). Respectively, 88.3% and 80% of the isolates from Dangassa and Nioro showed at least one substitution at these codons. However the prevalence of the particular pyrimethamine-resistance *Pfdhfr_51I*-*19R*-*108N* genotype reached 14.1% and 19.6% in Dangassa and Nioro-du-Sahel, respectively. Higher rate of codon substitution was observed in the sulfadoxine-resistance *Pfdhps* gene at positions 436, 437, 540, 581 and 613. These substitutions were more prevalent in Dangassa as compared to Nioro-du-Sahel (p value = 0.035). Up to 17.8% of the isolates from Dangassa against 7% for the isolates from Nioro-du-Sahel accumulated at least two (2) codon-substitutions were detected at these codons (Fig. 3).

The particular sulfadoxine-resistance haplotype *Pfdhps_437G*-*540E* was found in four isolates from Dangassa out of which only one exhibits the pyrimethamine-resistance *Pfdhfr_51I*-*19R*-*108N* genotype (the quintuple *Pfdhps_437G*-*540E*/*Pfdhfr_51I*-*19R*-*108N* substitution*)*.

The amodiaquine-resistance *Pfmdr1*-N86Y mutation was identified in 14.9% (6% *Pfmdr1*-Y86 + 8.8% *Pfmdr1*-N86/Y86) and 14.2% (8.9% *Pfmdr1*-Y86 + 8.3% *Pfmdr1*-N86/Y86) of the samples from Dangassa and Nioro-du-Sahel, respectively.

More importantly, the lumefantrine-reduced susceptibility *Pfmdr1_184F* mutation was found in 39.9% and 48.2% of samples from Dangassa and Nioro-du-Sahel, respectively. Only two (2) isolates from Dangassa exhibited the piperaquine-resistance *Exo*-*E415G* mutation. Such mutation was not found in Nioro. No artemisinins-resistance PGB was detected in the genotyped isolates. However, isolated codon substitutions constitutive of the artemisinin resistance PGB were found. Further information regarding the codon variation in studied genes is available in Additional file 1: Table S1.

**Fig. 4:**
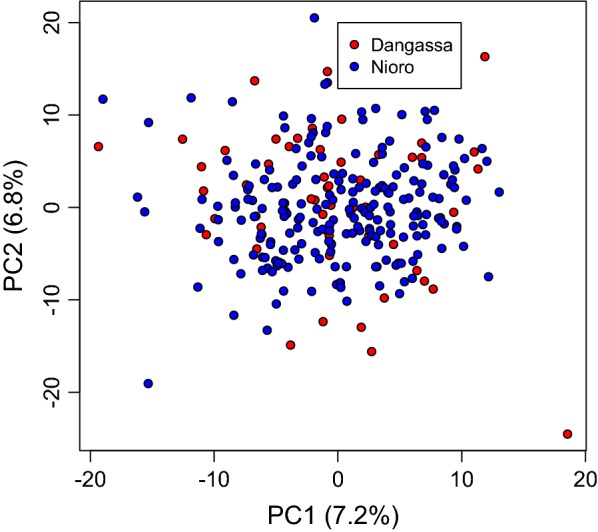
Plot Visualization of dot from Principal Component (PC) analysis of the 101 SNPs constitutive of barcode. This plot shows a wide distribution of the samples from both Dangassa and Nioro-du-sahel, with no aggregation of the dots

A principal component analysis of genotyping data from the Sanger 101 SNPs barcode showed very high *P. falciparum* genomic diversity in both Dangassa and Nioro-du-Sahel. However, no aggregation of specific *P. falciparum* genotype was found either in Dangassa or in Nioro (Fig. 4). Higher proportion of multiplicity of infection was observed in Dangassa using either COIL (p = 0.04) or Real McCOIL (p = 0.02) method based on the Sanger 101 SNPs barcode (Table 1).

**Table 1:** Multiplicity of infection in Dangassa and Nioro-du-Sahel patients, either with the MOI-COIL or the MOI-McCOIL

Index of multiplicity of infection	Dangassa (n = 214)		Nioro-du-sahel (n = 56)		Difference p
MOI-COIL					
1	139	65.3	44	83.0	
2	73	34.3	9	17.0	
3	1	0.5	0	0.0	0.043
MOI-McCOIL					
1	151	70.9	47	88.7	
2	61	28.6	6	11.3	
3	1	0.5	0	0.0	0.029

## Discussion

To protect the artemisinin molecules from resistance emergence in Africa and delay the spread of this resistance in the world, the WHO recommended ACT for uncomplicated malaria treatment in 2001 [4]. Such a strategy requires monitoring *P. falciparum* resistance to anti-malarial drugs in different geographical areas and adapting the appropriate drug combination. Moreover, the monitoring of the malaria parasite genetic variation will help to predict the dynamic of spread of the drug resistance through varying geographical areas. To assess molecular markers of anti-malarial drugs resistance and the genetic diversity in *P. falciparum* circulating isolates through the Malian territory, a passive case detection study was carried out in Dangassa and Nioro, two distinct epidemiological and eco-climatic zones in Mali. The revealed a high prevalence of the chloroquine resistance *Pfcrt_*76T in both localities. The *Pfcrt_*76T mutation was found in 64.3% in Dangassa and 42.5% in Nioro-du-Sahel. This is the first data report of anti-malarial drug resistance marker in Nioro, but a previous study has reported the prevalence of the chloroquine-resistance *Pfcrt_76T* mutation was of 85% in 2002 and 64.5% in 2003 in Kollé, a village sharing the same geographical area with Dangassa [31]. These observations suggest no decrease in the prevalence of chloroquine resistance marker in this region from 2002 to 2016 despite the official removal of chloroquine by the Malian NMCP since the years 2000s. Several studies in countries where chloroquine was removed reported substantial decrease in the prevalence of the chloroquine resistance marker suggesting a possible future reintroduction of this low-cost drug [43–46].

The data from this study revealed a very low prevalence of amodiaquine resistance-associated mutation *Pfmdr1*_86Y mutant parasite (two (2/214) in Dangassa and one (1/56) in Nioro-du-Sahel). The *Pfmdr1_86Y* mutation has been associated with amodiaquine resistance in Burkina Faso [47]. The *Pfmdr_86Y* mutant isolates was reported at the prevalence of 49.3% in Kolle, in 2002; however, the authors could not find association between this mutation and amodiaquine resistance in an in vivo assay [31]. Amodiaquine is one of most used anti-malarial drugs in Mali; it is associated with artemisinin in ACT and is also used in association with the SP in seasonal malaria control strategy (SMC) in children under five (5) years old in Mali.

Surprisingly, a high prevalence of *Pfmdr1_Y184F,* the lumefantrine-reduced susceptibility mutation was found in both Dangassa (39.9%) and Nioro-du-Sahel (48.2%). A higher prevalence of this mutation (53.8%) was reported in Senegal in 2013–2014 [48]. This finding was intriguing since lumefantrine has never been made available as a monotherapy in Mali. Lumefantrine has always been administrated in combination with artemether. Two possible scenarios may explain this finding. First, *P. falciparum* is less susceptible to the lumefantrine. This could impede on the efficacy of artemisinin-based strategy since artemether–lumefantrine is the most used ACT in Mali. Second, the *Pfmdr1_Y184F* mutation is not a good lumefantrine-resistance marker in Mali. Although several studies have reported an association between *Pfmdr1*_Y184F mutation and ex vivo reduced susceptibility to lumefantrine [49, 50], another in vivo studies could not establish this association [51]. Further in vitro investigations are needed to tease this observation apart.

The codon substitutions were assessed in the *Pfdhfr* and *Pfdhp* genes to identify SP resistant parasites, characterized by the inheritance of the *Pfdhfr*_51R-59N-108I/*Pfdhps*_437G-540E quintuple mutation. A high level of codon substitution was observed in these genes. The pyrimethamine-resistance Pfdhfr_51R-59N-108I genotype was found in 14.1% in Dangassa and 19.6% in Nioro-du-Sahel suggesting a decrease in the prevalence of this haplotype in the south of Mali as compared to the prevalence’s of 31.5% and 42.9% reported respectively 2002 and 2003 in Kolle [31]. The sulfadoxine resistance haplotype *Pfdhps*_437G-540E was found in only four isolates in Dangassa. Only one isolate presented the *Pfdhfr*_51R-59N-108I/*Pfdhps*_437G-540E quintuple substitution in Dangassa. SP is widely used in Mali for pregnant woman malaria chemoprophylaxis and seasonal malaria chemoprophylaxis in children under 5 years old. The data from this study suggest that the use of SP is still relevant in Mali, but previous reports suggest that such a malaria control strategy appears to increase the prevalence of SP-resistant parasite in Mali [32]. A very high rate of codon substitution was observed in these two genes (*Pfdhfr* and *Pfdhps*) in both Dangassa and Nioro-du-Sahel (Additional file 2).

No Pfarps10_127M-128Y/H-Pffd_193Y-Pfcrt_326S-356T-Pfmdr2_484I artemisinin resistance genetic background genotype was found during this study. Several mutations in P. falciparum kelch 13 protein propeller domain have been associated to resistance to artemisinins in Asia [28–30]. A genome wide association study revealed that these mutations always arise on the above specific genetic background [27]. Even though, no artemisinin resistance has been reported yet in Mali, [5, 52], a close monitoring for artemisinin resistance is essential. Further analysis of individual mutation constitutive of artemisinin resistance genetic background genotype may provide appropriate tool to predict artemisinin resistance emergence in Africa, where mutation in the Pfkelch13 propeller domain were not associated with artemisinin resistance [53].

*P*. *falciparum* genetic diversity is correlated with the level of transmission in different continental regions [54–56], and is influenced within regions by factors such as altitude, vector availability, urbanization, and malaria control strategies [57, 58]. In this study, it was hypothesized that the eco-climatic difference determines the malaria transmission variability between the two sites, and would impact the fitness of malaria parasites. Therefore, specific genetic variant of parasites could be selected in each site. Using the Sanger’s P. falciparum barcodes formed by concatenated genotypes at 101 SNPs across P. falciparum genome, the genetic diversity in P. falciparum circulating isolates was assessed in the study localities. These genotyping data showed a very high genetic diversity in P. falciparum isolates in both Dangassa and Nioro-du-Sahel supporting previous finding in Africa, even though those studies explored the msp1, msp2 or glurp genes size polymorphisms [59, 60]. No aggregation of specific P. falciparum genotype was observed at either study site. This finding may be due to the fact that the two sites are not geographically isolated. A higher complexity of P. falciparum infection was found in Dangassa, compared to Nioro-du-Sahel. These findings reflect the difference of transmission intensity between the two study sites. The complexity of infection is known to be associated with the intensity of malaria transmission [61, 62].

## Conclusions

This study revealed high diversity in P. falciparum circulating isolates in Mali. However no genetic population structure was found in either low or high transmission areas. No artemisinin resistance genetic background was found, but high prevalence of the lumefantrine resistance marker (Pfmdr1_Y184F genotype), the most used associated molecule to artemisinin in Mali, was detected. Further investigations are required to explore the lumefantrine resistance in Mali.

## Supplementary information

**Additional file 1: Table S1.** Baseline characteristics of the participants.** Table S2.** Clinical characteristics of participants.

**Additional file 2:** Codon substitutions in drug resistance genes.

## Data Availability

Public accession numbers for raw sequence data analysed are contained in SRA studies ERP000190 and ERP000199, as well as being accessible from the Pf3k project website (https://www.malariagen.net/projects/pf3k).

## Abbreviations

USTTB: University of Sciences, Technics and Technologies of Bamako
WACCBIP: West African Centre of Cell Biology of Infectious Pathogens
NGS: next-generation sequencing
PfCRT: *Plasmodium falciparum* chloroquine resistance transporter
PfDHFR: *Plasmodium falciparum* dihydrofolate-reductase
PfDHPS: *Plasmodium falciparum* dihydropteroate-synthetase
PfARPS10: *Plasmodium falciparum* Apicoplast Ribosomal Protein S10
PfFd: *Plasmodium falciparum* Ferredoxin
PfExo: *Plasmodium falciparum* exonuclease
PfMDR: *Plamodium falciparum* Multidrug resistance
COIL: complexity of infection likelihood
McCOIL: Method for the concurrent estimation of complexity of infection likelihood
NMCP: National Malaria Control Programme
ACT: artemisinin-based combination therapy
Artemisinins: artemisinin and its derivative drugs
WHO: World Health Organization
SNP: single nucleotid polymorphism
GWAS: Genome-Wide Association Study
PGB: parasite genetic background
LLINs: long lasting insecticide-impregnated nets
IPTp: intermittent preventive treatment of pregnant women
SP: sulfadoxine + pyrimethamine
RDT: rapid diagnostic test
HRP2: histidine rich-protein 2
pLDH: Plasmodium lactate dehydrogenage
i.m: intra muscular
WAG: whole genome amplification
