# Supplementary material for: A comprehensive analysis of drug resistance molecular markers and Plasmodium falciparum genetic diversity in two malaria endemic sites in Mali
Source: Malar J. 2019 Nov 12;18:361. doi: 10.1186/s12936-019-2986-5 (PMC6849310; doi:10.1186/s12936-019-2986-5)
Supplement: Supplementary file 1 — Additional file 1: Table S1. Baseline characteristics of the participants. Table S2. Clinical characteristics of participants. [file 12936_2019_2986_MOESM1_ESM.docx]

Additional file 1: Table S1

| **Characteristic** | **Dangassa (n=214)** | | **Nioro-du-sahel (n=56)** | | **Difference P** |
| --- | --- | --- | --- | --- | --- |
| **Age (years)** | 8 | (1-68) | 13 | (1-62) | 6x10^-6^ |
| **Weight (kg)** | 23 | (8-85) | 31 | (8-86) | 0.0001 |
| **Gender 2** | 120 | (56.6%) | 15 | (26.8%) | 0.0001 |
| **Ethnicity** |  |  |  |  |  |
| Malinke | 165 | 77.5 | 2 | 3.6 |  |
| Peulh | 17 | 8.0 | 8 | 14.3 |  |
| Sarakole | 12 | 5.6 | 13 | 23.2 |  |
| Senoufo | 2 | 0.9 | 1 | 1.8 |  |
| Bamabra | 2 | 0.9 | 23 | 41.1 |  |
| Bozo | 3 | 1.4 | 0 | 0.0 |  |
| Dogon | 0 | 0.0 | 1 | 1.8 |  |
| others | 12 | 5.6 | 8 | 14.3 | <3x10^-16^ |
| **Hb level (g/dL)** | 11.2 | (6.0-16.1) | 11.9 | (8.5-15.3) | 0.069 |
| **HbC** | 13 | 6.4 | 3 | 5.4 | 0.515 |
| **HbS** | 21 | 10.3 | 2 | 3.6 |  |
| **Blood group** |  |  |  |  |  |
| O+ | 89 | 41.8 | 24 | 42.9 |  |
| B+ | 46 | 21.6 | 12 | 21.4 |  |
| A+ | 44 | 20.7 | 8 | 14.3 |  |
| AB+ | 15 | 7.0 | 7 | 12.5 |  |
| B- | 7 | 3.3 | 1 | 1.8 |  |
| O- | 7 | 3.3 | 3 | 5.4 |  |
| missing data | 5 | 2.4 | 1 | 1.8 | 0.859 |

Additional file 1: Table S2

| Clinical Characteristics of malaria patients | Dangassa (n=214) | | Nioro-du-sahel (n=56) | | Difference P |
| --- | --- | --- | --- | --- | --- |
| **Fever in last 24 hrs** |  |  |  |  |  |
| Yes | 195 | 91.5 | 56 | 100 |  |
| No | 18 | 8.5 | 0 | 0 | 0.051 |
| **Parasite density (uL)** | Mean 17750 | Limits  (450-165000) | Mean 14980 | Limits (3863-75980) | 0.245 |
| **Hb level (g/dL)** | Mean 11.2 | Limits (6.0-16.1) | Mean 11.9 | Limits (8.5-15.3) | 0.069 |
| **Malaria severity** |  |  |  |  |  |
| Uncomplicated malaria | 119 | 55.9 | 41 | 73.2 |  |
| Severe malaria | 94 | 44.1 | 15 | 26.8 | 0.028 |
