# Supplementary material for: A comprehensive analysis of drug resistance molecular markers and Plasmodium falciparum genetic diversity in two malaria endemic sites in Mali
Source: Malar J. 2019 Nov 12;18:361. doi: 10.1186/s12936-019-2986-5 (PMC6849310; doi:10.1186/s12936-019-2986-5)
Supplement: Supplementary file 2 — Additional file 2: Codon substitutions in drug resistance genes. [file 12936_2019_2986_MOESM2_ESM.docx]

**Additional Files**

Drug resistance markers

| ***PfCrt 76*** | Dangassa | | Nioro-du-sahel | | Difference P |
| --- | --- | --- | --- | --- | --- |
|  | N | % | N | % |  |
| T | 85 | 45.9 | 20 | 37.7 |  |
| T/K | 34 | 18.4 | 4 | 7.5 |  |
| K | 66 | 35.7 | 29 | 54.7 | 0.025 |

| ***EXO 415*** | Dangassa | | Nioro-du-sahel | | Difference P |
| --- | --- | --- | --- | --- | --- |
|  | N | % | N | % |  |
| E | 206 | 96.7 | 53 | 94.6 |  |
| G | 1 | 0.5 | 0 | 0.0 |  |
| - | 6 | 2.8 | 3 | 5.4 | 0.566 |

| ***PfDhfr 51, 59, 108, 164,*** | Dangassa | | Nioro-du-sahel | | Difference P |
| --- | --- | --- | --- | --- | --- |
|  | N | % | N | % |  |
| -RNI | 67 | 31.5 | 21 | 37.5 |  |
| --NI | 43 | 20.2 | 4 | 7.1 |  |
| IRNI | 30 | 14.1 | 11 | 19.6 |  |
| NCSI | 17 | 8.0 | 10 | 17.9 |  |
| N**I | 10 | 4.7 | 3 | 5.4 |  |
| I-NI | 8 | 3.8 | 1 | 1.8 |  |
| N-SI | 7 | 3.3 | 1 | 1.8 |  |
| NRNI | 4 | 1.9 | 1 | 1.8 |  |
| -R*I | 4 | 1.9 | 0 | 0.0 |  |
| N-*I | 4 | 1.9 | 0 | 0.0 |  |
| N-NI | 4 | 1.9 | 0 | 0.0 |  |
| -**I | 2 | 0.9 | 1 | 1.8 |  |
| -HNI | 3 | 1.4 | 0 | 0.0 |  |
| -*NI | 2 | 0.9 | 0 | 0.0 |  |
| --*- | 1 | 0.5 | 0 | 0.0 |  |
| --*I | 1 | 0.5 | 0 | 0.0 |  |
| --N- | 0 | 0.0 | 1 | 1.8 |  |
| --SI | 1 | 0.5 | 0 | 0.0 |  |
| -CNI | 1 | 0.5 | 0 | 0.0 |  |
| -R-I | 1 | 0.5 | 0 | 0.0 |  |
| ***I | 0 | 0.0 | 1 | 1.8 |  |
| I-*I | 0 | 0.0 | 1 | 1.8 |  |
| I*NI | 1 | 0.5 | 0 | 0.0 |  |
| ICNI | 1 | 0.5 | 0 | 0.0 |  |
| N*SI | 1 | 0.5 | 0 | 0.0 | 0.213 |

| ***Pfdhps 436, 437, 540, 581, 613*** | Dangassa | | Nioro-du-sahel | | Difference P |
| --- | --- | --- | --- | --- | --- |
|  | N | % | N | % |  |
| -GKAA | 49 | 23.0 | 2 | 3.6 |  |
| -AKAA | 32 | 15.0 | 10 | 17.9 |  |
| SGKAA | 28 | 13.1 | 10 | 17.9 |  |
| SG-AA | 11 | 5.2 | 6 | 10.7 |  |
| AAKAA | 7 | 3.3 | 6 | 10.7 |  |
| SAKAA | 9 | 4.2 | 4 | 7.1 |  |
| -A-AA | 4 | 1.9 | 5 | 8.9 |  |
| -*KAA | 7 | 3.3 | 0 | 0.0 |  |
| -G-AA | 7 | 3.3 | 0 | 0.0 |  |
| -GKAS | 7 | 3.3 | 0 | 0.0 |  |
| *AKAA | 4 | 1.9 | 1 | 1.8 |  |
| *GKAA | 5 | 2.3 | 0 | 0.0 |  |
| AA-AA | 2 | 0.9 | 3 | 5.4 |  |
| AGKAA | 2 | 0.9 | 2 | 3.6 |  |
| SA-AA | 3 | 1.4 | 1 | 1.8 |  |
| *A-AA | 3 | 1.4 | 0 | 0.0 |  |
| -G-A* | 2 | 0.9 | 0 | 0.0 |  |
| -GEAA | 2 | 0.9 | 0 | 0.0 |  |
| -GKA* | 2 | 0.9 | 0 | 0.0 |  |
| *-KAA | 2 | 0.9 | 0 | 0.0 |  |
| AG-AA | 2 | 0.9 | 0 | 0.0 |  |
| AGKAS | 1 | 0.5 | 1 | 1.8 |  |
| ---AS | 1 | 0.5 | 0 | 0.0 |  |
| --KAA | 1 | 0.5 | 0 | 0.0 |  |
| -*KA* | 1 | 0.5 | 0 | 0.0 |  |
| -*KAS | 1 | 0.5 | 0 | 0.0 |  |
| -A-A* | 0 | 0.0 | 1 | 1.8 |  |
| -AKG- | 1 | 0.5 | 0 | 0.0 |  |
| -GK-A | 0 | 0.0 | 1 | 1.8 |  |
| *-K-- | 1 | 0.5 | 0 | 0.0 |  |
| *AKA* | 1 | 0.5 | 0 | 0.0 |  |
| *G-A* | 1 | 0.5 | 0 | 0.0 |  |
| *G-AA | 1 | 0.5 | 0 | 0.0 |  |
| *GEA* | 1 | 0.5 | 0 | 0.0 |  |
| AAKA* | 0 | 0.0 | 1 | 1.8 |  |
| AAKG- | 1 | 0.5 | 0 | 0.0 |  |
| AG-A* | 1 | 0.5 | 0 | 0.0 |  |
| AG-AS | 1 | 0.5 | 0 | 0.0 |  |
| AGKA- | 1 | 0.5 | 0 | 0.0 |  |
| FA-AS | 1 | 0.5 | 0 | 0.0 |  |
| S---- | 1 | 0.5 | 0 | 0.0 |  |
| S--AA | 0 | 0.0 | 1 | 1.8 |  |
| S-K-- | 1 | 0.5 | 0 | 0.0 |  |
| S-KAA | 0 | 0.0 | 1 | 1.8 |  |
| SA-A* | 1 | 0.5 | 0 | 0.0 |  |
| SGEAA | 1 | 0.5 | 0 | 0.0 |  |
| SGKA* | 1 | 0.5 | 0 | 0.0 |  |
| SGKAS | 1 | 0.5 | 0 | 0.0 |  |
| SGKG- | 1 | 0.5 | 0 | 0.0 | 0.035 |

| ***Pfmdr 186, 184, 1246*** | Dangassa | | Nioro-du-sahel | | Difference P |
| --- | --- | --- | --- | --- | --- |
|  | N | % | N | % |  |
| NFD | 85 | 39.9 | 27 | 48.2 |  |
| NYD | 42 | 19.7 | 11 | 19.6 |  |
| N*D | 26 | 12.2 | 2 | 3.6 |  |
| N-D' | 19 | 8.9 | 4 | 7.1 |  |
| YFD | 11 | 5.2 | 4 | 7.1 |  |
| *FD | 8 | 3.8 | 2 | 3.6 |  |
| **D | 4 | 1.9 | 1 | 1.8 |  |
| *-D | 4 | 1.9 | 0 | 0.0 |  |
| *YD | 3 | 1.4 | 0 | 0.0 |  |
| NF- | 2 | 0.9 | 1 | 1.8 |  |
| N-- | 0 | 0.0 | 2 | 3.6 |  |
| N*- | 2 | 0.9 | 0 | 0.0 |  |
| --D | 1 | 0.5 | 0 | 0.0 |  |
| -*D | 1 | 0.5 | 0 | 0.0 |  |
| -Y- | 1 | 0.5 | 0 | 0.0 |  |
| -YD | 0 | 0.0 | 1 | 1.8 |  |
| NY- | 1 | 0.5 | 0 | 0.0 |  |
| NYY | 1 | 0.5 | 0 | 0.0 |  |
| YFY | 1 | 0.5 | 0 | 0.0 |  |
| YYD | 1 | 0.5 | 0 | 0.0 |  |
| YYY | 0 | 0.0 | 1 | 1.8 |  |

| ***PGB (Pfarps10 127, 128, Pffd 193, Pfcrt 326, 356, Pfmdr2 484,*** | Dangassa | | Nioro-du-sahel | | Difference P |
| --- | --- | --- | --- | --- | --- |
|  | N | % | N | % |  |
| VDDNIT | 59 | 27.7 | 21 | 37.5 |  |
| VDDNTT | 41 | 19.2 | 12 | 21.4 |  |
| V-DNIT | 13 | 6.1 | 6 | 10.7 |  |
| V-DNTT | 13 | 6.1 | 3 | 5.4 |  |
| VDDN*T | 10 | 4.7 | 4 | 7.1 |  |
| -DDNTT | 10 | 4.7 | 0 | 0.0 |  |
| VDD-IT | 9 | 4.2 | 1 | 1.8 |  |
| -DDNIT | 8 | 3.8 | 0 | 0.0 |  |
| V-D-IT | 6 | 2.8 | 0 | 0.0 |  |
| VDD-*T | 5 | 2.3 | 0 | 0.0 |  |
| VDD-TT | 4 | 1.9 | 1 | 1.8 |  |
| -DDN*T | 4 | 1.9 | 0 | 0.0 |  |
| V-DN*T | 3 | 1.4 | 0 | 0.0 |  |
| VDYNTT | 3 | 1.4 | 0 | 0.0 |  |
| -DD-IT | 2 | 0.9 | 0 | 0.0 |  |
| -DYNIT | 2 | 0.9 | 0 | 0.0 |  |
| VD-NIT | 2 | 0.9 | 0 | 0.0 |  |
| VDDN-T | 1 | 0.5 | 1 | 1.8 |  |
| --DNIT | 0 | 0.0 | 1 | 1.8 |  |
| -D-N-- | 1 | 0.5 | 0 | 0.0 |  |
| -D-N*T | 1 | 0.5 | 0 | 0.0 |  |
| -D-NIT | 1 | 0.5 | 0 | 0.0 |  |
| -DY-*T | 1 | 0.5 | 0 | 0.0 |  |
| -DY-IT | 1 | 0.5 | 0 | 0.0 |  |
| -DY-TT | 1 | 0.5 | 0 | 0.0 |  |
| -DYN*T | 1 | 0.5 | 0 | 0.0 |  |
| *-DNIT | 0 | 0.0 | 1 | 1.8 |  |
| MDDN*T | 1 | 0.5 | 0 | 0.0 |  |
| V—NIT | 0 | 0.0 | 1 | 1.8 |  |
| V—NTT | 1 | 0.5 | 0 | 0.0 |  |
| V-D--- | 0 | 0.0 | 1 | 1.8 |  |
| V-D—T | 0 | 0.0 | 1 | 1.8 |  |
| V-D*TT | 1 | 0.5 | 0 | 0.0 |  |
| V-DN-T | 1 | 0.5 | 0 | 0.0 |  |
| VD—IT | 1 | 0.5 | 0 | 0.0 |  |
| VD-NTT | 0 | 0.0 | 1 | 1.8 |  |
| VD*N*T | 1 | 0.5 | 0 | 0.0 |  |
| VDD*IT | 0 | 0.0 | 1 | 1.8 |  |
| VDDN** | 1 | 0.5 | 0 | 0.0 |  |
| VDDSIT | 1 | 0.5 | 0 | 0.0 |  |
| VDY-IT | 1 | 0.5 | 0 | 0.0 |  |
| VDYN-T | 1 | 0.5 | 0 | 0.0 |  |
| VDYNIT | 1 | 0.5 | 0 | 0.0 |  |
